# Catestatin Exerts Direct Protective Effects on Rat Cardiomyocytes Undergoing Ischemia/Reperfusion by Stimulating PI3K-Akt-GSK3β Pathway and Preserving Mitochondrial Membrane Potential

**DOI:** 10.1371/journal.pone.0119790

**Published:** 2015-03-16

**Authors:** Eleonora Bassino, Sara Fornero, Maria Pia Gallo, Clara Gallina, Saveria Femminò, Renzo Levi, Bruno Tota, Giuseppe Alloatti

**Affiliations:** 1 Department of Life Sciences and Systems Biology, University of Torino, via Accademia Albertina 13, 10123, Torino, Italy; 2 Department of Clinical and Biological Sciences, University of Torino, Regione Gonzole 10, 10043, Orbassano (TO), Italy; 3 Department of Cell Biology, University of Calabria, Arcavacata di Rende (CS), 87030, Cosenza, Italy; 4 National Institute for Cardiovascular Research, via Irnerio 48, 40126, Bologna, Italy; Thomas Jefferson University, UNITED STATES

## Abstract

Catestatin (Cst) is a 21-amino acid peptide deriving from Chromogranin A. Cst exerts an overall protective effect against an excessive sympathetic stimulation of cardiovascular system, being able to antagonize catecholamine secretion and to reduce their positive inotropic effect, by stimulating the release of nitric oxide (NO) from endothelial cells. Moreover, Cst reduces ischemia/reperfusion (I/R) injury, improving post-ischemic cardiac function and cardiomyocyte survival. To define the cardioprotective signaling pathways activated by Cst (5 nM) we used isolated adult rat cardiomyocytes undergoing simulated I/R. We evaluated cell viability rate with propidium iodide labeling and mitochondrial membrane potential (MMP) with the fluorescent probe JC-1. The involvement of Akt, GSK3β, eNOS and phospholamban (PLN) cascade was studied by immunofluorescence. The role of PI3K-Akt/NO/cGMP pathway was also investigated by using the pharmacological blockers wortmannin (Wm), L-NMMA and ODQ. Our experiments revealed that Cst increased cell viability rate by 65% and reduced cell contracture in I/R cardiomyocytes. Wm, L-NMMA and ODQ limited the protective effect of Cst. The protective outcome of Cst was related to its ability to maintain MMP and to increase Akt^Ser473^, GSK3β^Ser9^, PLN^Thr17^ and eNOS^Ser1179^ phosphorylation, while treatment with Wm abolished these effects. Thus, the present results show that Cst is able to exert a direct action on cardiomyocytes and give new insights into the molecular mechanisms involved in its protective effect, highlighting the PI3K/NO/cGMP pathway as the trigger and the MMP preservation as the end point of its action.

## Introduction

In the last decade, several biologically active peptides derived from Chromogranin A (CgA), including Vasostatin-1 (VS-1) and Catestatin (Cst), have received attention as regulators of cardiovascular function [1–4]. CgA is highly conserved in the vertebrate secretory granules of both the diffuse neuroendocrine system and the heart itself, in which it colocalyzes with catecholamines and atrial and brain natriuretic peptides [5]. Initially used as a marker of several diseases such as neuro-endocrine tumors or neuro-degenerative dysfunctions [6], CgA recently emerged as a biomarker of cardiovascular pathologies such as essential hypertension [1–4], heart failure [7] and hypertrophic or dilatative cardiomyopathy [5]. The involvement of CgA in cardiovascular homeostasis is supported by its function as a pro-hormone. Among the products of CgA identified, Cst, initially described as a peptide exhibiting a catecholamine release-inhibitory action [1–4], displays several *in vivo* and *in vitro* effects on the heart and vasculature [8–11]. Cst exerts a vasodilator effect [8] and participates to blood pressure regulation: low Cst plasma levels have been measured not only in patients affected by essential hypertension, but also in their still normotensive offspring [12, 13]. Consistent with human findings [8, 12, 13], the severe hypertension, the dampened baroreflex sensitivity and the heart rate variability developed in knock-out mice for the CHGA gene can be rescued by the replacement with this peptide [14, 15]. Cardiac secretory granules containing CgA, CgB and Secretogranin 2 are present in murine heart. In particular, the fact that CgA is processed to Cst strongly suggests an autocrine/paracrine function of Cst within cardiac tissue [16]. Cst exerts a negative inotropic effect on the isolated rat heart and papillary muscle, both under basal conditions and in presence of β-adrenergic stimulation [9–10]. These effects were not due to a direct action on cardiomyocytes, but rather related to the activation of PI3K-Akt-eNOS pathway and NO release from endothelial cells [10]. At present, among the different actions of Cst on the heart, some need to be fully elucidated. The finding that Cst exerts a protective effect against I/R injury in isolated rat hearts [17, 18] has been questioned by other Authors, showing that Cst may also exert deleterious effects in these conditions [19]. In addition, our knowledge on the signaling pathways involved in the cardiovascular responses to CgA-derived peptides is, at present, only fragmentary. As typical high-affinity receptors have not been identified, the cellular processes upstream eNOS activation exerted by these peptides are still partially unknown [1–4]. In a recent work investigating the mechanism responsible for VS-1-mediated eNOS activation in endothelial cells, we showed that it involves a proteoglycans-phosphatidylinositol-4,5-biphosphate 3-kinase (PI3K)-dependent caveolae endocytosis as the initiating factor, providing an appealing novel signaling pathway expected for receptor-orphan peptides with membrane-interacting properties [20].

Although a number of experiments strongly suggests that endothelial cells play a fundamental role in mediating the effects of CgA-derived peptides on myocardium [10, 21], recent observations from our laboratory indicate that Cst is able to exert cardioprotective effects also via a direct effect, independent from endothelial cells, on isolated cardiomyocytes undergoing I/R [17]. Interestingly, this effect was attained at a very low concentration, comparable to the circulating concentrations of Cst in healthy humans [22]. These results reopen the question concerning the presence of specific receptors for Cst on cardiac cells, suggesting that the effects of CgA-derived peptides are, at least in part, independent from endothelial cells.

Therefore, besides to further confirm the direct cardioprotective effect exerted by Cst on isolated adult rat cardiomyocytes, the aim of the present study was to clarify the mechanisms and the signaling pathways involved in the action of this peptide. To this purpose, we evaluated the effects of Cst on cell viability and mitochondrial membrane potential stabilization on adult rat cardiomyocytes undergoing simulated I/R. The involvement of protein kinase B (Akt^Ser473^), glycogen synthase kinase 3β (GSK3β^Ser9^), endothelial nitric oxide synthase (eNOS^Ser1179^) and phospholamban (PLN^Thr17^) cascade was studied by immunofluorescence in the presence of the PI3K inhibitor wortmannin (Wm).

## Materials and Methods

### Animals

Experiments were performed on young adult CD®IGS rats (body weight 200–300 g), which were allowed *ad libitum* access to tap water and standard rodent diet. The animals received human care in compliance with the Guide for the Care and Use of Laboratory Animals published by the US National Institutes of Health (NIH Publication No. 85-23, revised 1996), and in accordance with Italian law (DL-116, Jan. 27, 1992). The scientific project was supervised and approved by the Italian Ministry of Health, Rome, and by the Committee on the Ethics of Animal Experiments of the University of Torino (session of 7/07/2010). Rats were anaesthetized by i.p. injection of Pentobarbital (Nembutal, 100 mg/Kg) and killed by stunning and cervical dislocation.

### Solutions and drugs

Tyrode standard solution contained (mM): 154 NaCl, 4 KCl, 2 CaCl_2_, 1 MgCl_2_, 5.5 D-glucose, 5 HEPES, pH adjusted to 7.4 with NaOH. Ca^2+^ free Tyrode solution contained (mM): 135 NaCl, 4 KCl, 1 MgCl_2_, 2 HEPES, 10 glucose, 10 butanedione monoxime, 5 taurine; pH adjusted to 7.4 with NaOH. Ischemic buffer (IB) solution contained (mM): 137 NaCl, 3.5 KCl, 0.88 CaCl2, 0.51 MgSO4, 5.5 D-Glucose, 4 HEPES, 10.2 deoxy-D-Glucose, 20 DL-lactic acid; pH adjusted to 6.5 with NaOH. Phosphate Buffer saline (PBS) solution contained (mM): 137 NaCl, 2.7 KCl, 10 Na_2_HPO_4_ • 2 H_2_O, 1.76 KH_2_PO_4_; pH adjusted to 7.4 with NaOH. All drug-containing solutions were prepared fresh before the experiments; except IB, all solutions were oxygenated (O_2_ 100%) before each experiment.

### Ventricular cells isolation

After sacrifice, the hearts were explanted, washed in modified Ca^2+^ free Tyrode solution and cannulated via the aorta. Hearts were perfused at a constant flow rate of 10 ml/min with Ca^2+^ free Tyrode solution with a peristaltic pump for approximately 5 min (37°C) to wash away the blood, then with 10 ml of Ca^2+^ free Tyrode supplemented with collagenase (0.3 mg/ml) and protease (0.02 mg/ml) and, finally, perfused and enzymatically dissociated with 30 ml of Ca^2+^ free Tyrode containing 50 μM CaCl_2_ and the same enzymatic concentration mentioned before. Ventricles were then separated from the atria, cut in small pieces and shaken for 10 minutes in 20 ml of Ca^2+^ free Tyrode solution in presence of 50 μM CaCl_2_, collagenase and protease.

### Experimental design

**Fig 1:**
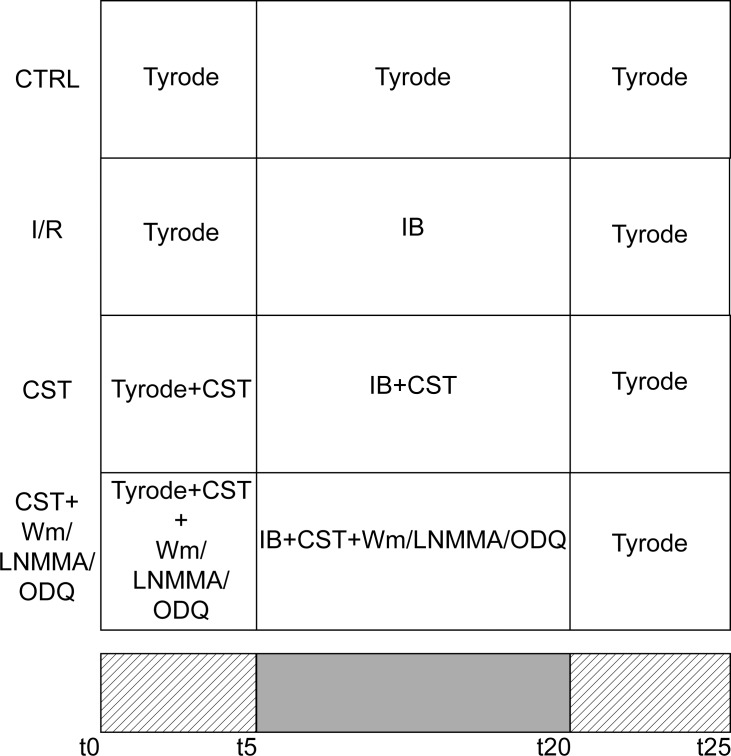
Experimental design. Control group (CTRL) cardiomyocytes were superfused/reperfused with Tyrode standard solution for 25 min. Ischemic/reperfused group (I/R): superfusion with Tyrode standard for 5 min, ischemic buffer (IB) for 15 min, and Tyrode standard (reperfusion) for 5 min. Ischemic + Cst group (Cst): superfusion with Tyrode standard + 5 nM Cst for 5 min, IB + 5 nM Cst for 15 min, and Tyrode standard alone for 5 min (reperfusion). Wortmannin (Cst + Wm), L-NMMA (Cst + L-NMMA) and ODQ (Cst + ODQ) groups: cardiomyocytes were treated as Cst group, in the presence of 100 nM Wm, 1 mM L-NMMA or 100 μM ODQ together with 5 nM Cst.

The experimental protocol for simulated I/R experiments is shown in Fig. 1. Control group (CTRL) cardiomyocytes were superfused with Tyrode standard solution for 25 min. In ischemic/reperfused (I/R) group, cardiomyocytes were superfused with Tyrode standard solution for 5 min, followed by IB perfusion for 15 min, and reperfused with Tyrode standard solution for 5 min. In Ischemic + Cst group (Cst), cardiomyocytes were treated with 5 nM Cst during the first 5 min of superfusion with Tyrode standard and along the entire IB treatment; reperfusion occurred in the presence of Tyrode standard solution alone. Wortmannin (Cst + Wm), L-NMMA (Cst + L-NMMA) and ODQ (Cst + ODQ) groups: cardiomyocytes were treated as Cst group, in the presence of 100 nM Wm, 1 mM NG-monomethyl-L-arginine (L-NMMA) or 100 μM 1H-[1,2,4]oxidiazolo[4,3-a]quinoxaline-1-one(ODQ) together with 5 nM Cst.

### Cell viability

Cell viability was evaluated by propidium iodide (PI, 10 μg/ml, Invitrogen) labeling. Images were acquired using a laser scanning confocal system (Fluoview 200, Olympus America, Melville, NY) with an Ar/Kr laser (488 and 568 nm) mounted on an inverted microscope (model IX70, Olympus) equipped with a ×20 UplanApo (NA 0.90). Confocal image acquisitions for each experimental condition were performed at the times t5, t20, and t25, as indicated in Fig. 1.

### Immunofluorescence and confocal microscopy

Cardiomyocytes were fixed for 30 minutes in 4% paraformaldehyde in 0.1 M phosphate buffer (PB), pH 7.3. After three washes with PBS, cells were incubated 20 minutes with 0.3% Triton and 1% bovine serum albumin (BSA, Sigma) in PBS and stained with the primary antibody 24 h at 4°C. Cover slides were washed twice with PBS and incubated 1h at room temperature with the secondary antibody. After two washes in PBS cover slides were mounted on standard slides with DABCO (Sigma) and observed after 24h under confocal microscope. We used a monoclonal anti-eNOS antibody (BD Biosciences, 1:50), a polyclonal anti-eNOS^Ser1179^ antibody (Invitrogen, 1:10), a polyclonal anti-GSK3β ^Ser9^ (Cell signaling 1:200), a polyclonal anti-Akt^Ser473^ antibody (Cell signaling 1:200), a polyclonal anti-PLN antibody (Cell signaling 1:500), PLN^Thr17^ (Cell signaling 1:200). Secondary antibodies employed for immunofluorescence experiments were Alexa Fluor 488 anti-mouse (Molecular Probes) for total eNOS and Cy3 anti-rabbit (Sigma) for P-eNOS^Ser1179^, P-PLN^Thr17^ and P-GSK3β ^Ser9^.

Confocal fluorimetric measurements were performed using an Olympus Fluoview 200 laser scanning confocal system (Olympus America Inc., Melville, NY, USA) mounted on an inverted IX70 Olympus microscope, equipped with a 60X Uplan FI (NA 1.25) oil-immersion objectives. Image processing and analysis were performed with ImageJ software (Rasband, W.S., U. S. National Institutes of Health, Bethesda, MA, http://rsb.info.nih.gov/ij/, 1997–2012).

### Mitochondrial membrane potential measurement

MMP was measured with a unique cationic dye of 5,5',6,6'-tetrachloro 1,1',3,3'-tetraethylbenzimidazolcarbocyaenina iodide (JC-1). Briefly, cells were stained with JC-1 (5 μg/ml) at 37°C for 30 min and then washed once with Tyrode standard solution. Then cells were treated according to the experimental protocol described above (Fig. 1). In living cells mitochondria appear red, due the aggregation of accumulated JC-1 (absorption/emission maxima of 585/590 nm), while the dye remains green in its monomeric form (absorption/emission maxima of 510/530 nm). In dead (apoptotic or necrotic) cells the dye is present almost only in the monomeric form (green fluorescence). For each cell the average intensity of green and red fluorescence was determined, and the ratio of JC-1 monomer (green) to aggregate (red) was calculated. An increase in this ratio was thus interpreted as an increase in mitochondrial membrane potential [23]. Image processing and analysis were performed with ImageJ software.

### Statistical analysis

Values are shown as means ± S.E.M. Statistical analysis was performed with either ANOVA followed by the Newman-Keuls multi-comparison test or the Student t test when appropriate. A p value < 0.05 was considered significant.

## Results

### Cst preserves cell viability and reduces contracture in I/R cardiomyocytes

Simulated I/R protocol markedly reduced the number of surviving cells respect to control (CTRL) group (18.1 ± 11.09% and 95.5 ± 3.1%, respectively). Cst 5 nM significantly preserved cell viability during reperfusion (63.45 ± 17.1%; p < 0.05; 5 experiments, at least n = 45 cells for each condition). The protective effect of Cst was completely blocked when cardiomyocytes were pre-treated with Wm 100 nM (21.0 ± 8.6%). The cardioprotective effect of Cst against I/R damage was further evaluated in terms of cardiomyocytes contracture. For each experimental condition, cell length was measured at t5 and t25 (the last corresponding to the end of the reperfusion phase). No significant variation of cell length was observed in CTRL group (from 111.8 ± 8.7 to 107.22 ± 9 μm, n = 18 cells). The marked reduction occurring during the reperfusion phase in the I/R group (from 95.0 ± 4.3 to 55.4 ± 3.7 μm; p < 0.001; n = 22 cells) was significantly reduced in Cst group (from 109.3 ± 3.2 to 104.6 ± 5.1 μm, n = 16 cells). In order to characterize the mechanism of action and the signaling pathways involved in the cardio-protection exerted by Cst, we performed additional experiments in the presence of pharmacological blockers of PI3K-Akt, NO and cGMP synthesis (Wm, L-NMMA and ODQ, respectively). All these drugs reduced the protective effect of Cst, and a remarkable contracture was observed again in cells of Cst + Wm (from 109.6 ± 6.9 to 79.7 ± 8.9 μm; n = 11 cells p < 0.01 respect to Cst group), Cst + L-NMMA (from 110.5 ± 8.4 to 95.6 ± 8.6 μm; n = 12 cells; p < 0.05 respect to Cst group) and Cst + ODQ (from 110.8 ± 4.7 to 91.5 ± 3.9 μm; n = 20 cells; p < 0.05 respect to Cst group) groups.

### The cardioprotective effect of Cst involves Akt, GSK3β and eNOS activation

**Fig 2:**
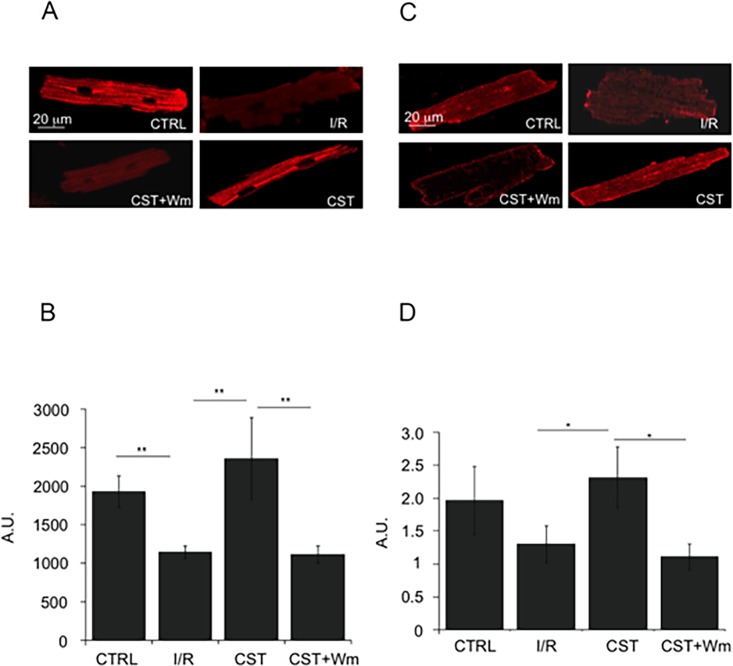
Cst preserves Akt and GSK3β activation during reperfusion. A) Fluorescence confocal images of cardiomyocytes labeled with anti phospho-Akt^473^ antibody in CTRL, I/R, Cst and Cst+Wm groups. B) Statistical analysis of the simulated I/R experiments. C) Fluorescence confocal images of cardiomyocytes labeled with anti phospho-GSK3β^Ser9^ antibody in CTRL, I/R, Cst and Cst+Wm groups. D) Statistical analysis of the simulated I/R experiments. For both Akt and GSK3β, fluorescence intensity (mean ± S.E.M. from at least 25 cells; n = 5 experiments) was expressed in arbitrary units (A.U.) for each treatment at the end of reperfusion phase. Statistical comparison was performed by one-way ANOVA followed by NM test for post hoc analysis (in this and the following figures: *p< 0.05; **p<0.01).

**Fig 3:**
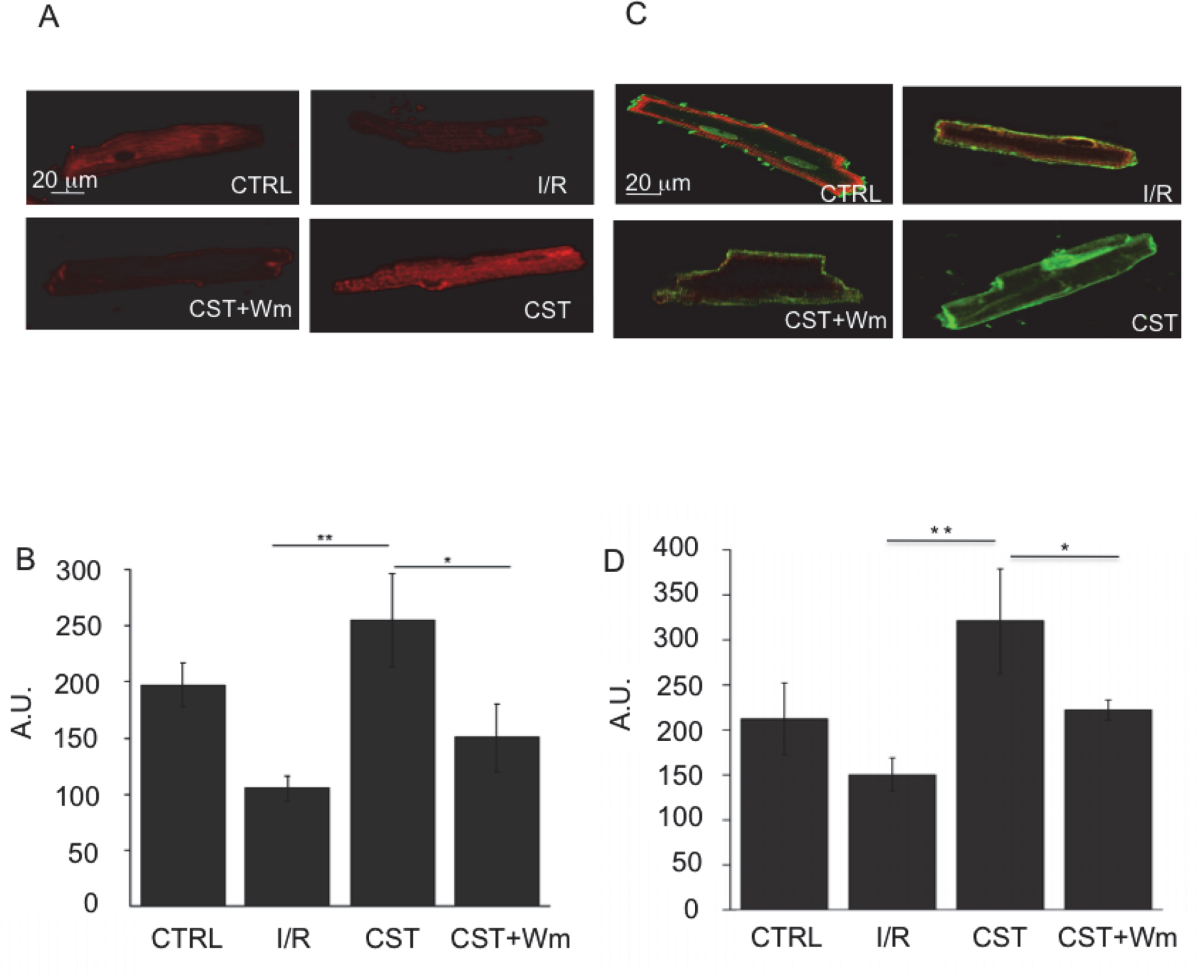
Cst maintains eNOS and PLN activation during reperfusion. A) Fluorescence confocal images of cardiomyocytes labeled with anti phospho-eNOS^1179^ antibody in CTRL, I/R, Cst and Cst+Wm groups. B) Statistical analysis of the simulated I/R experiments. C) Fluorescence confocal images of cardiomyocytes labeled with anti phospho-PLN^Thr 17^ (green fluorescence) antibody and with anti PLN antibody (red fluorescence) in CTRL, I/R, Cst and Cst+Wm groups. D) Statistical analysis of the simulated I/R experiments. For both eNOS and PLN, fluorescence intensity (mean ± S.E.M. from at least 25 cells; n = 5 experiments) was expressed as percentage respect I/R group for each treatment at the end of reperfusion phase.

To further investigate the role of PI3K-Akt/NO/cGMP pathway in the cardio-protection exerted by Cst, we performed immunofluorescence experiments on fixed cells exposed to simulated I/R, according to the protocol described in Fig. 1. Besides to verify whether Cst was able to modulate the phosphorylation of Akt and eNOS, we tested also the effect of Cst on GSK3β, another key enzyme involved in cardioprotection, included with Akt and eNOS in the reperfusion injury salvage kinase (RISK) pathway [24–27]. As indicated in Fig. 2 (A, B), I/R protocol caused a dramatic fall in the phosphorylation of Akt^Ser473^; Cst preserved the phosphorylation of this kinase, the level of which was comparable to that measured in control cells. The protective effect of Cst was abrogated in cells pretreated with Wm. Similar results were obtained for GSK3β^Ser9^: after I/R, Cst significantly increased the phosphorylation of GSK3β^Ser9^ in comparison to the I/R group (Fig. 2 C, D); also in this case, phosphorylation level was significantly reduced in the presence of Cst + Wm (p< 0.05). As shown in Fig. 3 (A, B), in cardiomyocytes undergoing I/R, Cst was also able to maintain phosphorylation of eNOS^Ser1179^ at a level comparable to that observed in CTRL cells, whereas in Cst + Wm samples this value was similar to that recorded in I/R group (p < 0.05).

### Cst maintains PLN activation

In order to study whether Cst is able to favour Ca^2+^ uptake by the sarcoplasmic reticulum under I/R conditions, we performed immunofluorescence experiments to test PLN^Thr17^ phosphorylation. We observed that, while in untreated ventricular cells PLN^Thr17^ phosphorylation level fell during reperfusion, Cst was able to maintain significantly higher levels of P-PLN during reperfusion (Fig. 3 C, D). The effect of Cst was completely abrogated by the treatment with Wm.

### Cst preserves mitochondrial membrane potential in I/R cardiomyocytes

**Fig 4:**
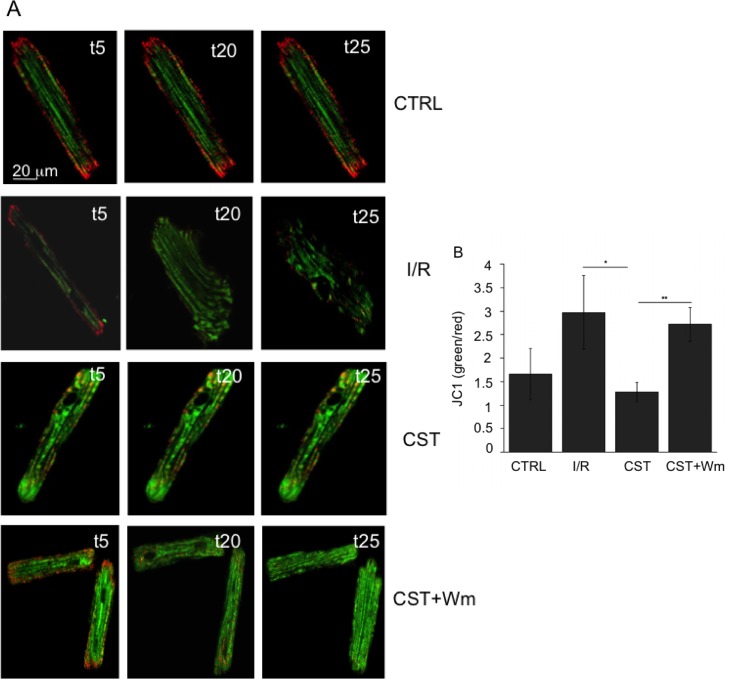
Cst preserves mitochondrial membrane potential in cardiomyocytes undergoing I/R. A) Fluorescence confocal images of cardiomyocytes labeled with JC-1 fluorescent probe at t5, t20 and t25 min of each experiment. B) Statistical analysis of green/red ratio (green: monomeric form; red: aggregate) of fluorescence variation. These values were referred to the end of the reperfusion phase. Results are presented as mean ± S.E.M. from at least 25 cells; n = 5 experiments.

To further investigate the potential protective effect of Cst against cell death induced by I/R, we finally evaluated its ability to maintain mitochondrial membrane potential in these conditions. To this purpose, we used the specific lipophilic cationic dye JC1, that exhibits a potential-dependent accumulation into the mitochondria displayed as a fluorescence emission shift from green (cytosolic) to red (mitochondrial) [23]. As indicated in Fig. 4, while in CTRL condition the green/red ratio of fluorescence variation was maintained constant throughout the entire time course of the experiment, in the I/R group the ratio began to increase at the end of the ischemic phase (t20), and further enhanced during reperfusion (t25). In cells treated with Cst the ratio of fluorescence variation was maintained constant for the entire experimental time course, in a fashion comparable to that of the CTRL group. Finally, the treatment of cells with Wm increased the green/red ratio to values comparable to those recorded in the case of the I/R group. As indicated in the bar graph (Fig. 4 B), Cst significantly reduced the green/red ratio of fluorescence respect to the I/R group, being able to rescue the physiological mitochondrial membrane potential values in myocardial cells undergoing I/R.

## Discussion

This study focused on the mechanisms responsible for the protective effect of Cst in isolated cardiomyoctes exposed to simulated I/R. Our findings indicate that Cst could limit I/R injury by stimulating key enzymes included in the RISK pathway, such as Akt, GSK3β and eNOS, and maintaining high PLN^Thr17^ phosphorylation levels, converging on MMP preservation. Moreover, the protection observed is primarily due to a direct effect on cardiomyocytes and does not necessarily depend on the endothelial effects of Cst. Interestingly, Cst-dependent cardioprotection was attained at a very low concentration, comparable to the circulating levels of this peptide found in healthy humans [22].

### The cardioprotective effect of Cst involves Akt, GSK3β and eNOS activation

Previous studies indicated that the inotropic effects of Cst are mediated by PI3K/Akt/eNOS/cGMP pathway [9–11]. Besides playing a key role in mediating intracardiac signaling involved in the control of contractile performance, this system is also involved in cardioprotection of I/R heart. In fact, among the signaling pathways underlying preconditioning, great attention has focused on the cGMP/protein kinase G (PKG) and on the RISK pathways, the last involving both Akt and the extracellular signal regulated kinase (ERK1/2). All these intracellular signaling cascades converge on the mitochondria *via* the modulation of several kinases, including GSK3β, Bax/Bad and the ε isoform of protein kinase C (PKC- ε) [24–27].

We thus hypothesized that the activation of these pathways are also responsible for the cardio-protective effect afforded by Cst. In line with this hypothesis, we show here that Cst is able to maintain high Akt, GSK3β and eNOS phosphorylation levels in isolated cardiomyocytes undergoing I/R. The blocking effect of Wm, L-NMMA and ODQ further confirms that the protective effect of Cst is due to the activation PI3K and eNOS, leading to cGMP synthesis.

In particular, the present study further proves the role of Akt in the cardioprotective effect of Cst against I/R-induced damage in the isolated rat heart [17, 18]. In contrast with these results, it has been reported that under certain conditions Cst does not activate Akt and exerts deleterious effects [19]. These conflicting results may be due to the different models of ischemia (*e*.*g*., global *vs*. regional ischemia) and/or the dose/duration of treatment used. Several agents, indeed, may be protective or deleterious when certain conditions have changed [27]. Interestingly, similarly to cardiomyocytes, anti-apoptotic Akt-dependent properties on endothelial cells have been recently described for Cst [28].

The ability of Cst to induce eNOS phosphorylation at the beginning of reperfusion is in agreement with studies performed on the isolated rat heart, in which both NO concentration and eNOS activity are increased during the first minutes of reperfusion [29]. The present study further confirms that GSK3β plays an important role in protection of cardiomyocytes exposed to I/R damage. Several recent studies support the notion that mitochondrial GSK3β plays a predominant role in myocyte necrosis after I/R injury. For instance, the threshold for mitochondrial permeability transition pores (mPTP) opening in response to reacting oxygen species (ROS) was significantly attenuated by both GSK3β inactivation or expression knockdown in cardiomyocytes [30]. The prevention of mPTP opening induced by Cst in the isolated I/R rat heart [17, 18] may thus be related to the ability to maintain the high GSK3β phosphorylation levels observed in our present experiments.

### Cst maintains PLN activation

By opening of mPTP, intracellular calcium overload occurring during reperfusion has a crucial role in mitochondrial dysfunction, hypercontracture and cell death [31]. We recently reported that in isolated rat I/R hearts, the administration of Cst during early reperfusion limited diastolic contracture and improved post-ischemic systolic function [17, 18]. Limited contracture is likely due to a reduced calcium overload resulting either from calcium extrusion and/or from increased re-uptake by sarco/endoplasmic reticulum Ca^2+^-ATPase (SERCA). While the former may reduce contractility, the latter tends to increase it. It has been suggested that RISK pathway activation may stimulate SERCA, thus enhancing calcium uptake into the sarcoplasmic reticulum, reducing calcium overload and preventing mPTP opening and cell death [27, 32].

We observed that Cst is able to maintain high PLN^Thr17^ phosphorylation levels in isolated cardiomyocytes undergoing I/R, comparable to those recorded in the pre-ischemic period. In cardiac cells, SERCA2a activity is modulated by PLN, the activity of which may be in turn regulated by phosphorylation in both Ser16 and/or Thr17 residues. While PLN^Ser16^ phosphorylation mainly accounts for the inotropic and lusitropic effects of β-adrenergic stimulation, PLN^Thr17^ phosphorylation is related to cardioprotection [33]. In particular, it has been pointed out that the phosphorylation of this residue is reduced after a prolonged period of ischemia (30 min), but it is maintained by brief periods of intermittent hypoxia, thus inducing cardioprotection [34]. In addition, it has been recently shown that activated Akt interacts and phosphorylates PLN at Thr^17^, thus improving Ca^2+^ handling and enhancing contractility [35].

### Cst preserves mitochondrial membrane potential in I/R cardiomyocytes

Mitochondrial damage is a determining factor in causing loss of cardiomyocyte function and viability during reperfusion injury. Major mechanisms of mitochondrial dysfunction include the long lasting opening of mPTPs and the consequent collapse of MMP [32]. Cardioprotective signaling pathways activated by both brief intermittent ischemic periods, such as preconditioning and postconditioning, and pharmacological agents converge on mitochondria to preserve their function after I/R. Our present observation that Cst was able to preserve MMP in cardiomyocytes undergoing I/R is in agreement with the previously reported ability of this peptide to reduce cell death and to avoid MMP dissipation due to oxidative stress in cultured H9c2 cells [18]. It is therefore reasonable that, as in the case of other cardioprotective agents, mitochondria represent the shared end point target of the Cst-activated signaling pathways, and preservation of their function thus corresponds to cell viability maintenance after I/R.

In conclusion, in the present work we demonstrated that Cst applied before and during an ischemic period exerts a protective action against I/R damage in isolated adult rat cardiomyocytes, by activating PI3K-Akt-GSK3β and eNOS pathways. Regarding in particular the role of NO in catestatin-dependent cardioprotection, we have to take into account that a limitation of the present model is the inability to evaluate the real contribution of autocrine NO production in the intact heart, in which the endothelial generation of NO is overwhelming.

Our study confirms the relevant role of Cst as a modulator of cardiovascular system, both under physiological and pathophysiological conditions. Thus, Cst may be considered a multifunctional peptide able to act at different levels, from the baroceptor and sympatho-cromaffin systems to the direct interaction with cardiomyocytes. In particular, our study highlights the importance of PI3K-Akt-GSK3β and eNOS pathways in the cardioprotective role of Cst, underlining its function in the stabilization of MMP and in the modulation of calcium signaling within cardiac cells undergoing ischemia and reperfusion.
